# Base Composition and Host Adaptation of the SARS-CoV-2: Insight From the Codon Usage Perspective

**DOI:** 10.3389/fmicb.2021.548275

**Published:** 2021-04-06

**Authors:** Ayan Roy, Fucheng Guo, Bhupender Singh, Shelly Gupta, Karan Paul, Xiaoyuan Chen, Neeta Raj Sharma, Nishika Jaishee, David M. Irwin, Yongyi Shen

**Affiliations:** ^1^Department of Biotechnology, Lovely Professional University, Phagwara, India; ^2^College of Veterinary Medicine, South China Agricultural University, Guangzhou, China; ^3^Guangdong Laboratory for Lingnan Modern Agriculture, Guangzhou, China; ^4^Department of Biochemistry, DAV University, Jalandhar, India; ^5^Department of Botany, St Joseph’s College, Darjeeling, India; ^6^Department of Laboratory Medicine and Pathobiology, University of Toronto, Toronto, ON, Canada; ^7^Banting and Best Diabetes Centre, University of Toronto, Toronto, ON, Canada; ^8^Key Laboratory of Zoonosis Prevention and Control of Guangdong Province, Guangzhou, China

**Keywords:** SARS-CoV-2, codon usage, base composition, codon pair usage, codon adaptation index, host adaptation

## Abstract

The novel severe acute respiratory syndrome coronavirus 2 (SARS-CoV-2) has been spreading rapidly all over the world and has raised grave concern globally. The present research aims to conduct a robust base compositional analysis of SARS-CoV-2 to reveal adaptive intricacies to the human host. Multivariate statistical analysis revealed a complex interplay of various factors including compositional constraint, natural selection, length of viral coding sequences, hydropathicity, and aromaticity of the viral gene products that are operational to codon usage patterns, with compositional bias being the most crucial determinant. UpG and CpA dinucleotides were found to be highly preferred whereas, CpG dinucleotide was mostly avoided in SARS-CoV-2, a pattern consistent with the human host. Strict avoidance of the CpG dinucleotide might be attributed to a strategy for evading a human immune response. A lower degree of adaptation of SARS-CoV-2 to the human host, compared to Middle East respiratory syndrome (MERS) coronavirus and SARS-CoV, might be indicative of its milder clinical severity and progression contrasted to SARS and MERS. Similar patterns of enhanced adaptation between viral isolates from intermediate and human hosts, contrasted with those isolated from the natural bat reservoir, signifies an indispensable role of the intermediate host in transmission dynamics and spillover events of the virus to human populations. The information regarding avoided codon pairs in SARS-CoV-2, as conferred by the present analysis, promises to be useful for the design of vaccines employing codon pair deoptimization based synthetic attenuated virus engineering.

## Introduction

The evolution of viruses is a conundrum for mankind. High mutation rates and host-shifts have culminated in multiple world-wide pandemics (Mackay and Arden, 2015; Luk et al., 2019). Recently, since December 2019, a viral outbreak by a novel coronavirus, named the severe acute respiratory syndrome coronavirus 2 (SARS-CoV-2), has emerged in Wuhan, China, and since then has rapidly spread throughout the world (Benvenuto et al., 2020a). In the recent past, two other coronavirus have resulted in major outbreaks, the severe acute respiratory syndrome (SARS) coronavirus (SARS-CoV) in 2002, which resulted in 8,096 cases of infection and 774 deaths, and the Middle East respiratory syndrome (MERS) coronavirus (MERS-CoV) in 2012, which infected 2,494 individuals and claimed 858 lives (Lu et al., 2020). Alarmingly, COVID-19, the disease caused by SARS-CoV-2, was declared to be the first coronavirus-related pandemic by WHO. The SARS-CoV-2 outbreak has already surpassed both the SARS-CoV and MERS-CoV outbreaks in terms of numbers of infected individuals and numbers of deaths (Lu et al., 2020), although the overall case-fatality rate for SARS-CoV-2 appears to be lower than those for both SARS-CoV and MERS-CoV (Wu and McGoogan, 2020).

Severe acute respiratory syndrome coronavirus 2 is a positive-stranded RNA virus that belongs to the genus *Betacoronavirus* within the family Coronaviridae (Benvenuto et al., 2020a). This newly emerged virus appears to be considerably distant from SARS-CoV (with around 79% identity) and MERS-CoV (around 50% identity) (Lu et al., 2020). Though, bats are believed to be the primary reservoirs for these coronaviruses, intermediate hosts are suggested to be involved before their final spillover events into humans (Zhou et al., 2020). Civets and dromedary camels have been reported to be the intermediate hosts for SARS-CoV and MERS-CoV, respectively (Mackay and Arden, 2015; Luk et al., 2019). The Malayan pangolin (*Manis javanica*) has been assumed to be the probable intermediate host for SARS-CoV-2 (Lam et al., 2020; Xiao et al., 2020), although a firm and definite conclusion is yet to be reached (Li et al., 2020).

Host adaptation is an extremely important aspect dictating the survival and reproductive prowess of viral pathogens (Butt et al., 2016; Chen et al., 2017). Given the degeneracy of the genetic code, the preferential usage of specific synonymous codons (codons encoding the same amino acid) leads to a codon usage bias in genes and genomes (Grantham et al., 1980). Codon usage patterns in viral genomes have been reported to be due to the impact of mutational pressure and selection for host translational efficiency (i.e., matching host codon bias) (Butt et al., 2016; Chen et al., 2017). Viruses, owing to their small genomes, largely depend on the cellular machinery of their hosts for processes such as replication and protein synthesis (Butt et al., 2016). Investigations into viral codon usage patterns, relative to their hosts, have proved to be instrumental in elucidating riddles concerning viral adaptation and evasion of host immune responses (Butt et al., 2016). Furthermore, exploring the potential role of intermediate hosts in the transmission route of viral pathogens has unraveled intriguing facets of viral spillover events (Butt et al., 2016; Chen et al., 2017).

Since the emergence of SARS-CoV-2, extensive research has been undertaken to decipher its genomic features and riddles of its transmission, evolutionary dynamics, epidemiology, and mode of infection (Alonso and Diambra, 2020; Benvenuto et al., 2020b; Chen et al., 2020; Dilucca et al., 2020; Gu et al., 2020; Kames et al., 2020; Lam et al., 2020; Lu et al., 2020; Malik et al., 2020; Wang et al., 2020; Wu et al., 2020; Xiao et al., 2020; Zhou et al., 2020). However, the complexities of codon usage by SARS-CoV-2 and its impact on the adaptation and fitness of this virus to human hosts have not yet been addressed. Accordingly, the present research endeavor has targeted base composition analysis and investigation of factors influencing the complex codon usage profile of this newly emergent virus and subsequent adaptation to the human host. Information obtained from this research, in combination with existing knowledge, promises to deepen our understanding of the basic biology, pandemicity, pathogenesis, and host adaptation of SARS-CoV-2.

## Materials and Methods

### Retrieval of Genomic Data

Genome sequences of 99 recently sequenced SARS-CoV-2, isolated from human (*Homo sapiens*) hosts, were retrieved from the GISAID repository^1^ (Supplementary Material 1) (as per data available on February 24, 2020; time of this work). Full genome sequence-based alignments were generated by employing MAFFT software (version 7.4.2) (Katoh and Standley, 2013) followed by editing with MEGA X software (version 10) (Kumar et al., 2018) according to the reading frames encoded by the reference SARS-CoV-2 genome (GenBank: MN908947). Five closely related Pangolin-CoVs and five Bat-CoVs isolated from the pangolins and bats, respectively, were also retrieved from GISAID and processed accordingly (Supplementary Material 1). Annotated coding sequences of the *H*. *sapiens* genome (GRCh38.p13) were fetched from NCBI GenBank. A total of 78 complete SARS-CoV genomes, isolated from human, civet and bat hosts, and 491 MERS-CoV genomes, representing human, dromedary camel, and bat hosts, were downloaded from NCBI GenBank database (Supplementary Material 1). The Genomic tRNA Database (GtRNAdb) (Chan and Lowe, 2009) was used to retrieve information regarding the isoacceptor tRNAs in *H*. *sapiens*.

### Estimation of Codon Usage Indices

Base compositional features of the viral coding sequences and estimates of codon usage, including relative synonymous codon usage (RSCU) and effective number of codons (ENC), were estimated employing CodonW (Ver. 1.4.2) software^2^ (Peden, 2000). Correspondence analysis, based on the RSCU data of the viral coding sequences, was also generated using CodonW.

### Neutrality Plot and Translational Selection Index

Neutrality plot analysis, an estimate of neutral evolution, was generated by plotting GC3 values (*x*-axis) of the viral genes against the respective GC12 values (*y*-axis) (Nasrullah et al., 2015; Butt et al., 2016).

Translational selection index (P2), an imperative estimate of translational selection, reflects the magnitude of interaction between a codon and its respective anticodon (Gatherer and McEwan, 1997). P2 was calculated as:

$$P\,2=\frac{W\,W\,C\,+\mathrm{SSU}}{\text{WWY}+\mathrm{SSY}}$$

where, W denotes the frequency of adenine (A) or uracil (U), S signifies the frequency of cytosine (C) or guanine (G), and Y reflects the frequency of cytosine (C) or uracil (U).

### Codon Adaptation Index

Codon adaptation index (CAI) efficiently depicts the probable expression levels of genes of interest with respect to the codon usage pattern of a highly expressed reference gene set (Puigbò et al., 2008; Butt et al., 2016). CAI values lie between 0–1 and higher CAI values of viral genes, with respect to the host codon usage pattern, have been suggested to reflect higher levels of viral adaptation to the host environment (Puigbò et al., 2008). The CAIcal server^3^ (Puigbò et al., 2008) was used to estimate CAI of the SARS-CoV-2 genes with respect to the *H*. *sapiens* host.

### Relative Dinucleotide Abundance and Relative Synonymous Codon Pair Usage

Relative abundance (P_*xy*_) of dinucleotides in SARS-CoV-2 and *H*. *sapiens* genes was computed as per the scheme suggested by Karlin and Burge (1995). The relative dinucleotide abundance was estimated as:

$$P_{x\,y}=\frac{f_{x\,y}}{f_{x}\,f_{y}}$$

where, f_*xy*_ and f_*x*_f_*y*_ refer to the observed and expected frequencies of the dinucleotide XY, respectively. Dinucleotides with P_*xy*_ > 1.25 were considered to be over-represented, whereas, dinucleotides with P_*xy*_ < 0.78 were inferred as under-represented (Kunec and Osterrieder, 2016).

The ratio of the observed to expected frequencies of a particular codon pair is commonly referred to as the relative synonymous codon pair usage (Kunec and Osterrieder, 2016). Codon pair scores were calculated as the natural logarithm of relative synonymous codon pair usage (Kunec and Osterrieder, 2016).

## Results

### Base Composition of SARS-CoV-2

Extensive analysis of the nucleotide composition of the viral coding sequences revealed a distinct trend of AU richness among the SARS-CoV-2 genomes. The average AU and GC contents (%) were observed to be 62.56 ± 0.05 and 37.44 ± 0.05, respectively. The mean compositions (%) of the nucleotides A (28.15 ± 0.04) and U (34.41 ± 0.06) were found to be significantly higher than G (17.87 ± 0.02) and C (19.57 ± 0.03) (*P < 0.01*). An analysis of the RSCU revealed that a majority (20 out of 26 codons) of the preferentially employed codons (RSCU > 1) were AU rich (Table 1). The average composition (%) of AU3 (70.02 ± 0.05) was found to be significantly higher than GC3 (29.98 ± 0.05) (*P < 0.01*). It was also evident that 25 out of the 26 preferentially employed codons ended with A/U nucleotides (Table 1).

**TABLE 1:** Relative synonymous codon usage (RSCU) patterns of SARS-CoV-2 in comparison with its host *Homo sapiens*.

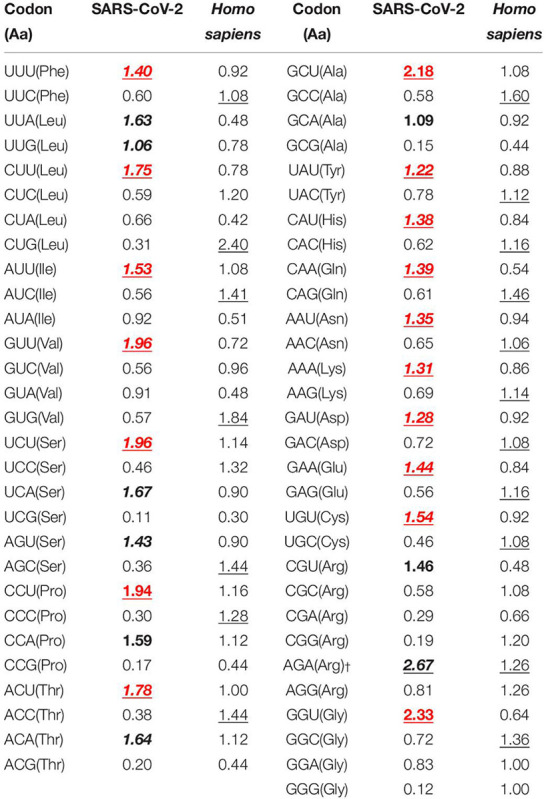

*Preferentially employed codons (RSCU > 1.00) in SARS-CoV-2 are marked in bold; AU rich preferentially employed codons in SARS-CoV-2 are marked in italics; Most preferred codons in SARS-CoV-2 and H. sapiens are underlined; Most preferred codons in SARS-CoV-2 displaying antagonism with H. sapiens are marked in red color; Coincident codon of SARS-CoV-2 and H. sapiens are marked with †.*

*Aa, amino acid.*

### Estimates of the Codon Usage in SARS-CoV-2

**FIGURE 1:**
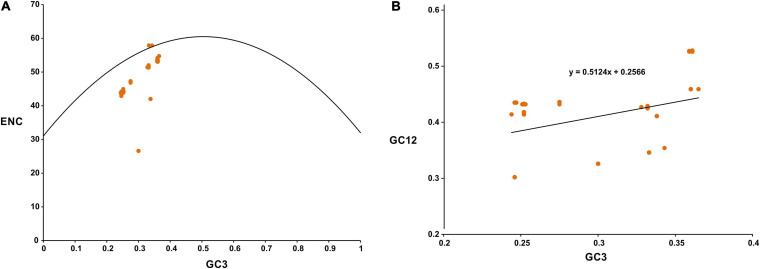
**(A)** GC3-ENC plot for SARS-CoV-2. Viral genes analyzed are marked as orange colored circles. ENC plot curve is indicated by the bell-shaped solid line. **(B)** Neutrality plot for SARS-CoV-2. Viral genes analyzed are marked as orange colored circles.

The average ENC of the viral coding sequences was found to be 46.16 ± 5.93. An analysis of the GC3 versus ENC plot for SARS-CoV-2 revealed that while certain viral genes clustered on the continuous ENC plot curve (or close to it), others fell well below the curve (Figure 1A). It has been suggested that the continuous ENC plot curve indicates the expected codon usage of genes if GC compositional constraints alone account for the codon usage bias (Wright, 1990). It was interesting to note that a group of coding sequences in the GC3 versus ENC plot for SARS-CoV-2 (Figure 1A) presented low ENC values (below 30) and behaved as outliers. After a thorough scrutiny it was observed that the group of coding sequences represented ORF7b of SARS-CoV-2 which was only 129 bp. It has been suggested that coding sequences less than 300 bp might behave as outliers due to small sizes in codon usage analysis (Xiang et al., 2015). A comprehensive analysis of the neutrality plot revealed that the slope of the regression line (Figure 1B) was around 0.5124, signifying a 51.24% influence of the compositional constraint on the viral coding sequences. The average translational selection index (P2) value of the SARS-CoV-2 coding sequences was found to be 0.43 ± 0.04, which suggested that apart from mutational bias, natural selection had a role in influencing the SARS-CoV-2 codon usage pattern.

Correspondence analysis, a multivariate statistical method, was performed on the RSCU data of SARS-CoV-2 to identify the determinants of codon usage variation employing CodonW. High significant correlations of GC and GC3 contents with Axes 1 and 2 of the RSCU data, the two major principle axes for the separation of genes, revealed a pronounced impact of compositional constraint on the SARS-CoV-2 genomes (Table 2). Strong correlation of the ENC with GC (*r* = 0.49, *P* < 0.01) and GC3 (*r* = 0.48, *P* < 0.01) contents reinforced the governing impact of compositional bias. Axes 1 and 2 of the RSCU data were found to correlate significantly with CAI of the SARS-CoV-2 genomes (Table 2), thus, depicting an undeniable influence of natural selection. Significant correlation of CAI with ENC (*r* = 0.81, *P* < 0.01) further signified the impact of natural selection in shaping the codon usage signatures of SARS-CoV-2. It is evident from Table 2 that the length of the viral coding sequences correlated significantly with Axes 1 and 2 of the RSCU data. Furthermore, factors such as hydropathicity index {GRAVY [positive GRAVY (hydrophobic), negative GRAVY (hydrophilic)]} and aromaticity of the encoded viral gene products also correlated significantly with Axis 1 of the RSCU data (Table 2).

**TABLE 2:** Correlation analysis (Spearman’s rank correlation) of various codon usage indices of SARS-CoV-2 with the principle axes of separation of the genes to Axes 1 and 2 of the RSCU data.

**Codon usage indices**	**Axis1 (RSCU)**	**Axis 2 (RSCU)**
**A**	0.14**	−0.41**
**G**	−0.32**	0.42**
**C**	−0.44**	0.36**
**U**	0.21**	0.12**
**ENC**	−0.59**	−0.33**
**GC**	−0.49**	0.42**
**GC3**	−0.58**	0.37**
**Length**	0.13**	0.11**
**Gravy**	−0.32**	0.05
**Aromo**	−0.16**	–0.08
**CAI**	0.74**	−0.60**

**Statistically significant at *P < 0.01*. Aromo, aromaticity of the viral gene product; ENC, effective number of codons; GRAVY, grand average hydropathicity score; Length, length of viral protein coding sequence; CAI, codon adaptation index.

### Relative Dinucleotide Abundance in SARS-CoV-2

**FIGURE 2:**
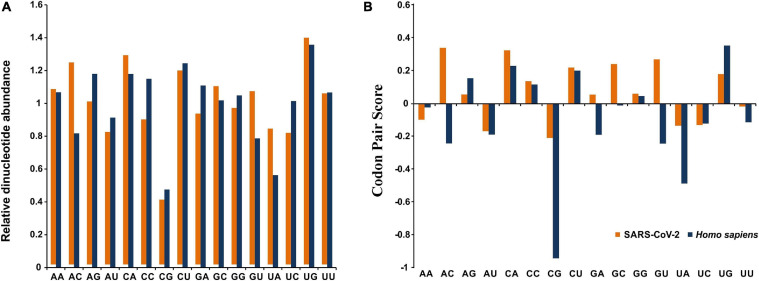
**(A)** Relative dinucleotide abundance for SARS-CoV-2 in comparison with its host *Homo sapiens*. **(B)** Dinucleotide bias at the codon–codon interface for SARS-CoV-2 in comparison with its host *Homo sapiens*.

Robust analysis of the relative dinucleotide abundance in SARS-CoV-2 revealed that UpG (1.38 ± 0.02) and CpA dinucleotides (1.27 ± 0.02) were over-represented (Figure 2A). Dinucleotide ApC (1.23 ± 0.02) and CpU (1.18 ± 0.03) were found to be marginally preferred in SARS-CoV-2 (Figure 2A). An analysis of RSCU revealed that the UpG containing codons, such as UUG and UGU, and CpA containing codons, like UCA, ACA, CCA, GCA, CAU, and CAA, were preferred (RSCU > 1.00) in SARS-CoV-2 (Table 1). These observations correlated well with the over-representation of these concerned dinucleotides. The CpG dinucleotide was observed to be highly under-represented (0.39 ± 0.01) in SARS-CoV-2, a pattern consistent with its host *H. sapiens* (Figure 2A). Codons, such as GCG, UCG, CGC, CGA, CGG, CCG, and ACG, containing the CpG dinucleotide were noted to be under-represented (RSCU < 0.60) (Table 1). The dinucleotides UpA (0.82 ± 0.03), ApU (0.81 ± 0.02), and UpC (0.82 ± 0.01) were found to be marginally avoided in SARS-CoV-2 (Figure 2A).

### Patterns of Codon Pair Usage in SARS-CoV-2

An extensive analysis of the relative synonymous codon pair usage, of the 3,721 (61 × 61) codon pairs (excluding stop:stop and stop:sense codon pairs), revealed that 1,530 codon pairs were over-represented (positive codon pair score), whereas, 2,187 were under-represented (negative codon pair score) in SARS-CoV-2 (Supplementary Material 2). The codon pair CGG-CGG, coding for the amino acid pair Arg–Arg, was noted to be most over-represented with a codon pair score of 4.27. On the other hand, the codon pair AAU-AUA, encoding the amino acid pair Asn-Ile, displayed the lowest codon pair score of −4.56 and was inferred to be most avoided.

Dinucleotide patterns NNU-GNN, NNC-ANN, NNC-UNN, and NNA-CNN, representing the dinucleotides UpG, CpA, CpU, and ApC, respectively, were predominant at codon-codon junctions in SARS-CoV-2 (Figure 2B). The NNU-GNN (representing the UpG dinucleotide at the codon pair junction) was found to be most prevalent (11.24%) among the over-represented codon pairs.

Dinucleotide patterns NNC-GNN, NNU-ANN, and NNA-UNN, representing the dinucleotides CpG, UpA, and ApU, respectively, were noted to be highly avoided at codon pair junctions in SARS-CoV-2 (Figure 2B). The NNC-GNN pattern was noted to be most abundant (10.06%) among the under-represented codon pairs, suggesting strong suppression of CpG dinucleotides at the codon-codon interface of the SARS-CoV-2 genomes.

### Investigating the Patterns of SARS-CoV-2 Adaptation to Human Hosts

#### Antagonistic Codon Usage Patterns of SARS-CoV-2 Toward Human Host

Detailed RSCU analysis and profiling the most preferred codons (for each amino acid) in SARS-CoV-2 and *H*. *sapiens* revealed a distinct trend of antagonism between the viral and human codon usage patterns (Table 1). Seventeen out of the eighteen most preferred codons in SARS-CoV-2 were found to exhibit antagonism with *H*. *sapiens*, whereas the codon AGA (coding for Arg), was the only one to display coincidence (Table 1).

### Most Preferred Codons in SARS-CoV-2 and Human Isoacceptor tRNAs

Identification of the most preferred codons (for each amino acid) in SARS-CoV-2 and the most abundant isoacceptor tRNAs in human cells revealed that 6 out of the 18 most preferred codons in SARS-CoV-2, namely, GCU, CCU, ACU, UCU, CUU, and AUU (coding for the amino acids Ala, Pro, Thr, Ser, Leu, and Ile, respectively), optimally matched with the respective most abundant isoacceptor tRNAs in human hosts (Table 3).

**TABLE 3:** The most preferred codon, for each amino acid, in SARS-CoV-2 and iso-acceptor tRNAs in *Homo sapiens*.

**Amino acids**	**Most preferred codons in SARS-CoV-2**	**tRNA isotypes in *Homo sapiens***
Ala	GCU	**AGC (22)**, GGC (0), CGC (4), UGC (8)
Gly	GGU	ACC (0), GCC (14), CCC (5), UCC (9)
Pro	CCU	**AGG (9)**, GGG (0), CGG (4), UGG (7)
Thr	ACU	**AGU (9)**, GGU (0), CGU (5), UGU (6)
Val	GUU	AAC (9), GAC (0), CAC (11), UAC (5)
Ser	UCU	**AGA (9)**, GGA (0), CGA (4), UGA (4), ACU (0), GCU (8)
Arg	AGA	ACG (7), GCG (0), CCG (4), UCG (6), CCU (5), UCU (6)
Leu	CUU	**AAG (9)**, GAG (0), CAG (9), UAG (3), CAA (6), UAA (4)
Phe	UUU	AAA (0), GAA (10)
Asn	AAU	AUU (0), GUU (20)
Lys	AAA	CUU (15), UUU (12)
Asp	GAU	AUC (0), GUC (13)
Glu	GAA	CUC (8), UUC (7)
His	CAU	AUG (0), GUG (10)
Gln	CAA	CUG (13), UUG (6)
Ile	AUU	**AAU (14)**, GAU (3), UAU (5)
Tyr	UAU	AUA (0), GUA (13)
Cys	UGU	ACA (0), GCA (29)

*Most abundant iso-acceptor tRNAs in *Homo sapiens* matching the most preferred codons of SARS-CoV-2 are marked in bold.*

### Adaptive Efficacy of SARS-CoV-2 in Human Host in Comparison With SARS-CoV and MERS-CoV

**FIGURE 3:**
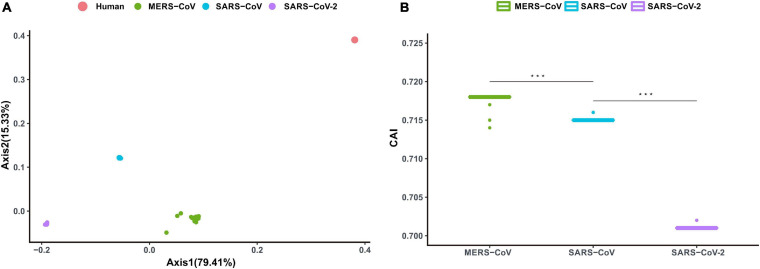
**(A)** Correspondence analysis depicting Axis 1 and Axis 2 of the RSCU data for MERS-CoV, SARS-CoV, SARS-CoV-2, and their human host. Dots representing MERS-CoV, SARS-CoV, and SARS-CoV-2 are marked in green, blue, and purple, respectively. Dots signifying human coding sequences are marked in red. **(B)** Codon adaptation index (CAI) values for MERS-CoV, SARS-CoV, and SARS-CoV-2. Mann–Whitney U Rank Sum Test was used to compare the average of the CAI values pertaining to the different sets of viruses. *P < 0.05; **P < 0.01; ***P < 0.001.

The magnitude of adaptive efficacy and associated fitness of the recently emerged SARS-CoV-2 to the human niche was explored in light of the adaptation exhibited by other relevant and notorious coronaviruses associated with severe pneumonia and past outbreaks, namely SARS-CoV and MERS-CoV. A correspondence analysis based on the RSCU data of the concerned viral genomes (SARS-CoV-2, SARS-CoV, and MERS-CoV) and the human genome led to the formation of four discrete clusters, with the viral clusters falling close to each other (Figure 3A). It was intriguing to note that the cluster formed by MERS-CoV was closest to that formed by the human genome (spatial Mahalanobis distance of 169.41), followed by the SARS-CoV cluster (spatial Mahalanobis distance of 206.71) (Figure 3A). Interestingly, the cluster formed by SARS-CoV-2 was observed to be most distant from the human genome cluster (spatial Mahalanobis distance of 1618.40) (Figure 3A). Axis 1 of the RSCU data (contributing 79.41% of the variation) was found to display a strong correlation with CAI values of the respective viral genomes (*r* = 0.87, *P* < 0.01). CAI of the viral genes, calculated with respect to host codon usage patterns, provides insights into the degree of viral adaptation to the host cellular environment. The average CAI value for SARS-CoV-2 with respect to *H*. *sapiens* was found to be 0.701 ± 0.04, which was significantly lower (*P* < 0.01) than for both MERS-CoV (0.718 ± 0.05) and SARS-CoV (0.715 ± 0.04) (Figure 3B). Our observations indicate a relatively lower adaption of the newly emerged SARS-CoV-2, in contrast to SARS-CoV and MERS-CoV, to human cellular systems.

### Role of the Intermediate Host in the SARS-CoV-2 Spillover Event to the Human Population

**FIGURE 4:**
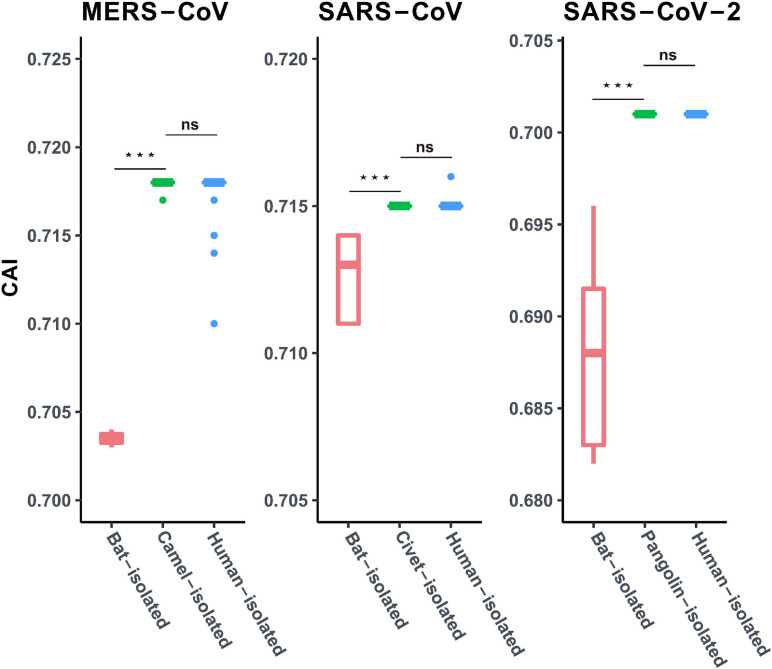
Codon adaptation index (CAI) values for MERS-CoV, SARS-CoV, and SARS-CoV-2, isolated from different hosts, calculated with the human coding sequences as reference. Red, green, and blue boxes represent viral isolates from the bat, corresponding intermediate host (dromedary camel for MERS-CoV, civet for SARS-CoV, and pangolin for SARS-CoV-2) and human host, respectively. Mann–Whitney U Rank Sum Test was employed to compare the mean of the CAI values pertaining to the different sets of viruses. *P < 0.05; **P < 0.01; ***P < 0.001. ns, not significant.

In order to investigate the potential role of the intermediate host in the SARS-CoV-2 transmission event, we compared the CAI values of human isolated SARS-Cov-2, closely related Pangolin-CoVs and Bat-CoVs, employing human codon usage patterns as a reference for calculation. Our results displayed similar patterns of adaptation to the human cellular system for SARS-Cov-2 and closely related Pangolin-CoVs (*P* < 0.01) (Figure 4). However, the CAI values of the closely related Bat-CoVs were noted to be significantly lower (*P* < 0.01) (Figure 4) in comparison to SARS-Cov-2 and Pangolin-CoVs, when assessed in reference to human codon usage patterns. We further analyzed the patterns of adaptation of SARS-CoV and MERS-CoV genomes isolated from bats (primary reservoir for both of the concerned coronaviruses), intermediate hosts (civets in the case of SARS-CoV and dromedary camels in the case of MERS-CoV) and the terminal hosts human beings. Our results revealed that the CAI values of SARS-CoV and MERS-CoV (estimated employing human codon usage patterns) isolated from bats were significantly lower than the CAI values of the respective viral isolates representing the intermediate and human hosts (*P* < 0.01) (Figure 4). Thus, the trend of similar adaptation patterns for SARS-Cov-2, SARS-CoV, and MERS-CoV isolates from intermediate and human hosts was found to be consistent.

## Discussion

The recent emergence of the novel SARS-CoV-2 has posed a serious threat to global public health (Benvenuto et al., 2020a). The rapid spread and transmission of this virus demands an investigation into its adaptive fitness and patterns of acclimatization into the newly introduced human host. The present research effort was undertaken to comprehensively explore the complex codon usage profile of SARS-CoV-2, relative to the human host, and confers meaningful knowledge pertaining to viral adaptation to the human niche.

Our analysis revealed that the coding sequences of SARS-CoV-2 had a high average of ENC (ENC = 46.16 ± 5.93), which signified a pattern of low codon usage bias (Figure 1A). It has been suggested that a virus with low codon usage bias might be more flexible and able to adapt and maintain its survival cycle in a broad range of hosts with different codon usage signatures (Jenkins and Holmes, 2003; Luo et al., 2020).

Thorough our analysis of a GC3-ENC plot of SARS-CoV-2 we revealed that while certain viral genes fell on or close to the continuous ENC plot curve, others clustered well below the curve (Figure 1A). It has been suggested that if codon usage of a gene is governed only by compositional bias, then it would lie on or above the continuous ENC plot curve, whereas, the clustering of genes well below the curve signifies the impact of other factors such as natural selection, in addition to compositional constraint (Wright, 1990). Thus, it was evident that apart from compositional constraint others factors such as translational selection also influenced the codon usage behavior of SARS-CoV-2. A neutrality plot (Figure 1B) revealed that the degree of compositional constraint influence on the coding sequences was around 51.24%, which further supports our conclusion (Nasrullah et al., 2015; Butt et al., 2016). Strong correlations of GC and GC3 contents of the viral coding sequences with the two major axes of separation of the RSCU data reinforce the pronounced impact of compositional bias (Table 2). It has been suggested that the translational selection index (P2) > 0.50 signifies a major role of translational selection acting on the concerned genes (Gatherer and McEwan, 1997). An average P2 value of 0.43 ± 0.04 for the SARS-CoV-2 coding sequences signified that, apart from compositional constraint, translational selection had a considerable influence on the viral codon usage patterns. Apart from compositional bias and translational selection, factors including length of the viral protein coding sequence, hydropathicity index (GRAVY) and aromaticity of the viral protein products also display significant correlations with the RSCU data (Table 2). Thus, codon usage signatures of the SARS-CoV-2 appeared to be result of various imperative factors including compositional bias, natural selection, hydropathicity and aromatic character of the viral gene products, and the lengths of the viral coding sequences, with compositional bias displaying the most crucial impact. Similar cases inferring compositional bias as the major contributor of codon usage variations have been previously reported for SARS-CoV (Gu et al., 2004) and MERS-CoV (Chen et al., 2017).

Severe acute respiratory syndrome coronavirus 2 has an AU rich genome with a distinct preference toward the usage of AU rich codons over their GC rich counterparts (Table 1). Similar trends have been observed in the closely related SARS-CoV (Gu et al., 2004) and MERS-CoV (Chen et al., 2017) genomes. SARS-CoV-2 was found to strongly abstain from using the dinucleotides CpG and UpA, a pattern consistent with the host *H*. *sapiens* (Figure 2A). Similar patterns of CpG and UpA dinucleotide avoidance was evident at the codon-codon interface of the viral genomes (Figure 2B). CpG and UpA under-representation is a trademark of vertebrate genomes (Kunec and Osterrieder, 2016). Avoidance of the CpG dinucleotide is an established feature of RNA viruses (Kunec and Osterrieder, 2016). It has been suggested that the unmethylated CpGs of viral pathogens are recognized by the host intracellular pattern recognition receptor Toll like receptor 9 (TLR9) to stimulate an immune response against the pathogens (Dorn and Kippenberger, 2008; Kunec and Osterrieder, 2016). Thus, suppression of CpG dinucleotides in SARS-CoV-2 appears to be a strategy to evade human immune response. Under-representation of UpA dinucleotides in SARS-CoV-2 might be a reflection of its effort to hone translational efficacy by reducing the risk of nonsense mutations, mRNA degradation, and error-prone translation associated with UpA abundance (Karlin and Burge, 1995). Interestingly, a significant share of the over-represented (52.22%) and under-represented codon pairs (56.29%) in SARS-CoV-2 matched with host *H. sapiens*, which indicates adaptation toward enhanced robustness of SARS-CoV-2 to the human cellular environment.

The rapid spread of COVID-19 necessitates an urgent need of safe and effective vaccines to combat the pandemic. Analysis of codon usage and information regarding under-represented codons and codon pairs in viral genomes offer scopes toward the development of live-attenuated vaccines employing the synthetic attenuated virus engineering approach (Coleman et al., 2008). Synthetic attenuated virus engineering involves recoding and synthesis of a viral genome in a way that preserves the wild-type amino acid sequence but rearranges existing synonymous codons to create a sub-optimal arrangement of codon pairs that are typically under-represented (Coleman et al., 2008). The identification and profiling of under-represented codons and codon pairs containing the CpG and UpA dinucleotides (Table 1 and Figure 2) in SARS-CoV-2 genomes through extensive codon usage analysis, as executed in the present study, promises to be useful in guiding deoptimization of codons and codon pairs for viral attenuation and vaccine development. Such applications have been successfully implemented in the development of live-attenuated vaccines against poliovirus (Coleman et al., 2008), human respiratory syncytial virus (Le Nouën et al., 2014), influenza virus (Mueller et al., 2010), dengue virus (Shen et al., 2015), Lassa virus (Cai et al., 2020), and enterovirus A71 (Tsai et al., 2019).

Severe acute respiratory syndrome coronavirus 2 was found to display antagonistic codon usage patterns with the human host (Table 1). Similar trends of antagonism have previously been seen in the Marburg virus (Nasrullah et al., 2015) and the hepatitis A virus (Sanchez et al., 2003) with the human host. In contrast, poliovirus was found to display a complete coincidence (Mueller et al., 2006) and Zika virus (Butt et al., 2016) has been reported to exhibit a mixture of antagonism and coincidence with the codon usage patterns of the human genome. It has been suggested that coincident patterns of codon usage between a virus and its host facilitates translational efficiency, whereas, antagonism facilitates proper folding of the viral proteins, although the efficacy of translation might be reduced (Hu et al., 2011).

Most of the preferred codons in SARS-CoV-2 coding sequences use suboptimal isoacceptor tRNAs from human cells (Table 3). A similar pattern of suboptimal tRNA isotype recognition use has been previously reported for the Nipah virus (Khandia et al., 2019). It has been suggested that the usage of suboptimal isoacceptor host tRNAs during the initial phase of an infection might facilitate slow but precise translation, which yields the synthesis of accurate and properly folded viral proteins (Khandia et al., 2019).

The CAI of viral genes, estimated with respect to host codon usage patterns, has been proposed to be an effective index of the degree of viral adaptation to a host’s cellular environment (Puigbò et al., 2008). Lower CAI value for SARS-CoV-2, in comparison to MERS-CoV and SARS-CoV (Figure 3B), estimated with respect to human codon usage patterns, signifies moderately adapted potential and fitness of this recently emerged pathogen to human cellular system and seems to be in agreement with its long incubation time (between 1 and 14 days), milder infective consequences and relatively lower case-fatality rates of between 3 and 4% (as reported by WHO), in contrast to the enhanced infective manifestations and higher case-fatality rates of 34.40 and 9.56% reported for MERS-CoV and SARS-CoV (Wu and McGoogan, 2020). Considering the fact that this virus has already spread globally, and that human populations are highly susceptible, there exists a possibility that SARS-CoV-2 might enhance its adaptive finesse to human cells through ongoing processes of adaptive evolution, leading to further risks for transmission and imminent outbreaks.

Viral transmission across different hosts and associated cross-species jumps are puzzling events in the intricate transmission dynamics of these viruses (Smith et al., 2009). Intermediate hosts are believed to play a crucial role in the viral spillover events to human populations. There have been many instances of the involvement of intermediate hosts in viral pandemics and epidemics (Smith et al., 2009; Parrish et al., 2015). It has been suggested that the involvement of an intermediate host, or a series of hosts, facilitates viral transmission from its natural reservoirs and acts as a platform to hone adaptive finesse of the viral pathogens before the final spillover to the human population (Parrish et al., 2015). In this context, it is interesting to note that the viral isolates from the pangolins (Pangolin-CoVs) display highly similar patterns of adaptation with SARS-CoV-2, in the human cellular niche (as evident from similar CAI values) (Figure 4). Similar trends were observed for other coronaviruses such as SARS-CoV and MERS-CoV that have infected human populations, where the CAI values of the viral isolates from their respective intermediate hosts matched those isolated from humans, thus signifying similar adaptive patterns (Figure 4). In contrast, the respective viral isolates from primary bat reservoirs displayed significantly weaker adaptation (as evident from lower CAI values) to human host systems (Figure 4). These results suggest that pangolins have the potential to act as the intermediate host for SARS-CoV-2 and that the Pangolin-CoVs represents a potential future threat to public health (Lam et al., 2020; Xiao et al., 2020). However, more systematic research efforts and exhaustive long-term monitoring of SARS-related coronaviruses in pangolins and other related animals would be necessary to draw a final inference about the mysterious intermediate host of SARS-CoV-2.

Multiple studies have been targeted at the investigation of codon usage patterns of SARS-CoV-2 till date (Alonso and Diambra, 2020; Dilucca et al., 2020; Gu et al., 2020; Kanduc, 2020; Malik et al., 2020; Saha et al., 2021). Recently, Tort et al. (2020) reported a close relationship of SARS-CoV-2 with Bat-CoVs in comparison to SARS-CoV, MERS-CoV, and other coronaviruses isolated from civets and ferrets, based on a comparative codon usage analysis. The study indicates toward the involvement of bats as probable primary reservoirs for SARS-CoV-2 (Tort et al., 2020). However, in the present analysis based on codon usage, apart from bats we have also explored the potential association of pangolins in the transmission route of SARS-CoV-2 to humans. The present research pertaining to the codon usage patterns of SARS-CoV-2, together with its transmission dynamics involving intermediate hosts, and facets of its adaptation to its newly introduced human population promises to significantly contribute toward the elucidation of its infective manifestations and rapid global spread, thus bolstering current efforts on pandemic preparedness. Extensive knowledge pertaining to codon usage and the profiling of preferred and avoided codons and codon pairs in the viral genomes have been effective in synthetic attenuated virus engineering toward the development of live-attenuated vaccines (Burns et al., 2006; Coleman et al., 2008). Deoptimized viral genes and genomes, with under-represented codon pairs, have been reported to exhibit decreased replicative fitness and low protein expression levels without major alterations that evoke host immune responses (Burns et al., 2006; Coleman et al., 2008). The identification and profiling of under-represented codon pairs in SARS-CoV-2, as executed in the present analysis, might prove beneficial toward the rational development of safe attenuated vaccines and combat the COVID-19 pandemic.

## Data Availability Statement

The original contributions presented in the study are included in the article/Supplementary Material, further inquiries can be directed to the corresponding author/s.

## Author Contributions

YS conceived, designed, and supervised the study. AR, FG, BS, SG, KP, XC, NS, and NJ generated the data. YS, AR, and FG analyzed the data. YS, DI, and AR wrote and prepared the manuscript. All authors have read and agreed to submission of the manuscript.

## Conflict of Interest

The authors declare that the research was conducted in the absence of any commercial or financial relationships that could be construed as a potential conflict of interest.

## Footnotes

1 http://www.GISAID.org

2 http://www.molbiol.ox.ac.uk/cu

3 http://genomes.urv.cat/CAIcal/RCDI/

## Supplementary Material

Supplementary Material 1 Information pertaining to the accession numbers and hosts (isolation source) of the SARS-CoV-2, SARS-CoV, and MERS-CoV genomes used in the present study.

Supplementary Material 2 Codon pair scores of the 3,721 (61 × 61) codon pairs (excluding stop:stop and stop:sense codon pairs) that were analyzed for SARS-CoV-2.
